# The Effects of Waste Cement on the Bioavailability, Mobility, and Leaching of Cadmium in Soil

**DOI:** 10.3390/ijerph18168885

**Published:** 2021-08-23

**Authors:** Xiuming Ding, Junfeng Wang, Qing Huang, Shan Hu, Yuejun Wu, Luya Wang

**Affiliations:** 1 College of Ecology and Environment, Hainan University, Haikou 570228, China; dingxiuming42@gmail.com (X.D.); hushan2000@126.com (S.H.); wuyuejun1992@gmail.com (Y.W.); wangluya4025@gmail.com (L.W.); 2 State Key Laboratory of Marine Resource Utilization in South China Sea, Hainan University, Haikou 570228, China; 3 College of Civil and Transportation Engineering, Shenzhen University, Shenzhen 518061, China

**Keywords:** waste cement, cadmium, adsorption, immobilization, bioavailability, leaching

## Abstract

Waste cement is a construction and demolition waste produced from old buildings’ demolition and transformation. In recent years, the recycling of recycled concrete is limited to the use of recycled aggregate, and the research on the utilization of waste cement in waste concrete is scarce. This study explored the effective application of waste cement for the adsorption of cadmium (Cd^2+^) from an aqueous solution and the bioavailability and immobility of Cd^2+^ in soil. Results showed that the maximum adsorption capacities of ordinary Portland cement(OPC) paste, fly ash cement (FAC) paste, and zeolite cement (ZEC) paste for Cd^2+^ were calculated to be 10.97, 9.47, 4.63 mg·g^−1^, respectively. The possible mechanisms for Cd^2+^ adsorption in the solution by waste cement mainly involve precipitation by forming insoluble Cd^2+^ compounds in alkaline conditions, and ion exchange for Cd^2+^ with the exchangeable calcium ions in waste cement, which were confirmed by XRD and SEM. Results from diethylene triaminepentaacetic acid (DTPA) extraction and toxicity characteristic leaching procedure (TCLP) implied reduction of the Cd^2+^ mobility. DTPA-extractable Cd^2+^ decreased by 52, 48 and 46%, respectively, by adding 1% OPC, FAC and ZEC. TCLP-extractable Cd^2+^ decreased by 89.0, 80.3, and 56.0% after 1% OPC, FAC, and ZEC treatment, respectively. BCR analyses indicate that OPC, FAC, and ZEC applications increased the percentage of Cd^2+^ in residual fraction and induced a high reduction in the acid-soluble Cd^2+^ proportion. The leaching column test further confirmed a reduction in Cd^2+^ mobility by waste cement treated under continuous leaching of simulated acid rain (SAR). Therefore, waste cement exhibited a significant enhancement in the immobilization of Cd^2+^ under simulated acid rain (SAR) leaching. In summary, the application of alkaline waste cement could substantially remove Cd^2+^ from wastewater and reduce Cd^2+^ mobility and bioavailability in contaminated soil.

## 1. Introduction

Cd^2+^ is widely distributed throughout the world due to its high toxicity and long biological half-life [1]. Cd^2+^ cause harmful effects on human and plants due to its high mobility and toxicity [2,3,4]. Soil Cd^2+^ contamination has become a serious environmental problem with the development of mining exploration, metallurgy industry, solid waste disposal, paints pigments, and wastewater irrigation [5,6]. Acid rain in southern China makes the Cd^2+^ in the soil unstable and spreads to the surrounding environment through surface runoff or leaching to groundwater [7]. The restoration of Cd^2+^ contaminated soil to minimize the risks to human and ecological health needs attention.

Soil remediation by in situ immobilization is a promising technique that stabilizes potentially toxic elements in the soil by adding natural or artificial materials. It is a rapid, cost-effective, and environmentally friendly remediation technology [8,9]. In recent years, many amendments have been developed for immobilization of Cd^2+^ in soil, such as lime, cement, fly ash, organic amendments, clay minerals, phosphate minerals, and industrial and agricultural by-products [10,11,12,13,14]. The primary mechanisms of immobilization include surface absorption, ion exchange, precipitation, and formation of stable complexes [15]. The addition of these amendments decreases the availability of heavy metals in contaminated soil and increases immobilization. However, large-scale decontamination of medium and/or heavily polluted mining areas can be too costly. Therefore, it is an important challenge to select an effective and cost-competitive amendment for remediation of heavy metal contaminated soil.

Although soil is a sink of a heterogeneous mixture of various components, the binding of Cd^2+^ in the soil is diverse due to its composition. Therefore, probing the physicochemical forms of the Cd^2+^, such as their bioavailability, mobility, and leaching, are necessary to understand their environmental behavior. Cd^2+^ bioavailability, mobility, and leaching in soil depend on several factors such as pH, cation exchange capacity (CEC), organic matter, soil texture, and clay content. Soil pH is the most significant and major factor that can influence the behavior of Cd^2+^ mobility and bioavailability in soil [16]. Therefore, the important characteristics of an amendment are to have the potential to increase soil pH and improve soil acid neutralization capacity.

Among all possible modifications, Portland cement has proven as the most widely used amendment on acid soils, due to its low cost, high efficiency, simple operation, and availability [17]. Previous studies have indicated that the immobilization mechanism of cement is precipitation or coprecipitation by metal ions in the form of calcium hydroxide, calcium silicate hydrate, and hydrated calcium aluminate [18]. However, there are some disadvantages in cement immobilization, such as the increase in soil volume, soil hardening, capacity enhancement, and soil alkalinity after remediation. The pH value of the remediation soil is generally greater than 12, which is a highly alkaline environment and is not conducive to the long-term stability of heavy metal fixation [18]. Such detriments of cement immobilization are raising questions and bringing about an increasing demand from leading researchers to develop potential soil additives that have similar mitigation properties but are relatively mild alternatives to cement, especially those that are more effective, widely applicable, and less costly, to ameliorate contaminated soil.

With the rapid urbanization of China, massive construction and renovation activities have resulted in a large amount of construction and demolition waste. Consequently, construction and demolition waste has become one of the largest solid wastes in China [19]. Concrete and bricks are the basic components of construction, which are inert materials and are generally consider least damaging to the environment [20,21,22]. Waste cement is one of the main components of construction and demolition waste, which is a kind of highly hydrated Portland cement. Waste cement has the advantage of the high pH of Portland cement, but since it has been highly hydrated, it will not cause soil hardening after application. Therefore, it offers the opportunity to apply on large scale for the remediation of heavy metal in mining cites. In recent years, some studies in the literature have mentioned the application of construction and demolition waste as a remediation tool for soil heavy metals [23,24,25,26]. For example, Poorahong [24] observed that concrete particles (0.5–1 mm) containing cement paste, sand, and rock could increase the pH to slightly alkaline of acidic wastewater containing heavy metals, and remove Cr, Cu^2+^, Ni, Pb, and Zn^2+^ with high efficiency through a combination of adsorption and precipitation. In recent examples, the Cd^2+^ adsorption mechanism on cement pastes and successful Cd^2+^ removal from Cd^2+^ solution and Cd^2+^ contaminated soils were demonstrated by Damrongsiri [23]. Results revealed that the high pH and high acid-neutralizing capacity of cement paste could enhance Cd^2+^ immobilization and increase the capacity to neutralize acid input. However, no information is available about the application of demolition waste addition into Cd^2+^ polluted soil to examine its mobility, bioavailability, and leaching.

Cement paste could improve the heavy metal adsorption capacity of soil and soil acid-neutralizing capacity; thus, it can be considered as an alternative immobilizing agent for heavy metal contaminated soil when applied to the mining areas in southern China, where the heavy metal pollution zone overlaps with the areas affected by acid rain. Hence, it was hypothesized that whether the different kinds of cement paste (ordinary Portland cement (OPC) paste, fly ash cement (FAC) paste, and zeolite cement (ZEC)) commonly used in construction activities may be highly effective for immobilization of heavy metal in acid-contaminated soil in mining areas. The main objectives of this study were (1) to assess the potential of waste cement amendment to reduce Cd^2+^ bioavailability and soil acidity; (2) to explore the possible mechanisms of waste cement amendment for Cd^2+^ immobilization, and; (3) to evaluate effects of waste cement amendment on Cd^2+^ distribution in soil, leaching characteristics and its mobility in contaminated soil.

## 2. Materials and Methods

### 2.1. Waste Cement Production

**Figure 1:**
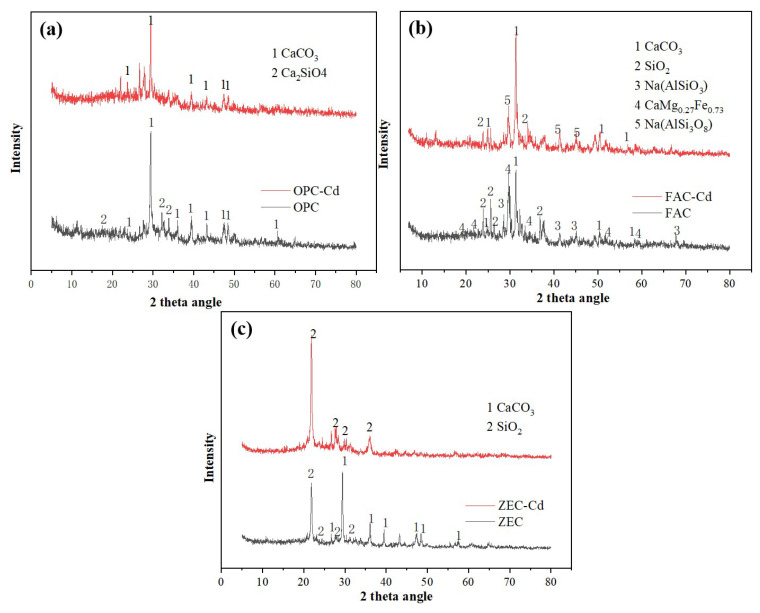
X-ray diffraction spectra for (**a**) cement paste (OPC), (**b**) fly ash cement (FAC) paste, and (**c**) zeolite cement (ZEC) paste before and after adsorption of Cd^2+^.

**Figure 2:**
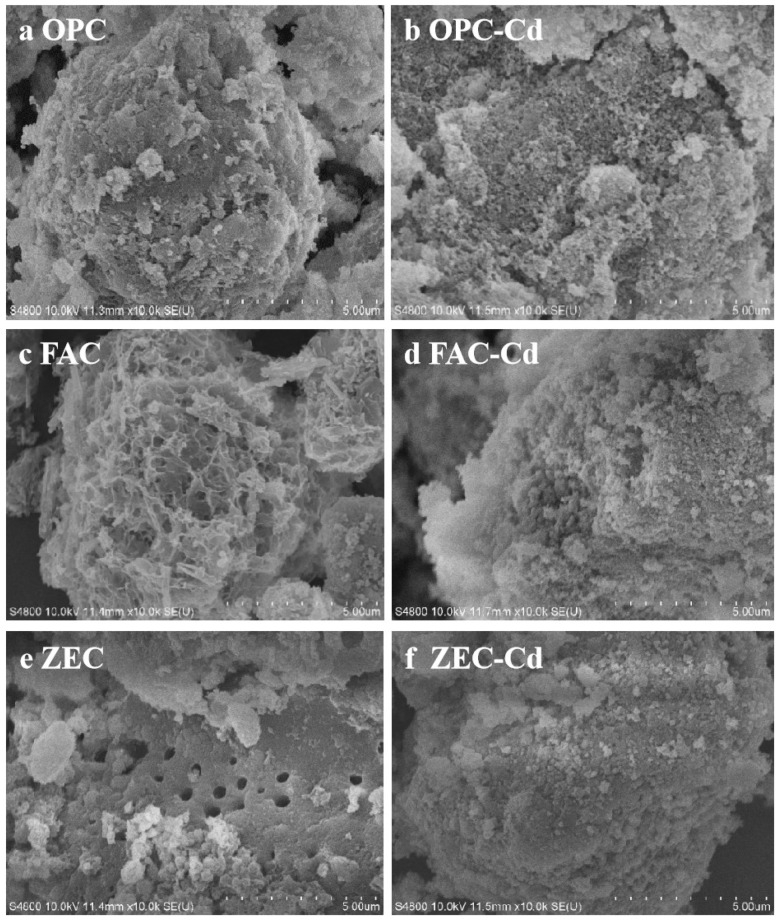
SEM images of Cd^2+^ adsorption with cement paste (OPC), fly ash cement (FAC) paste, and zeolite cement (ZEC) paste: (**a**,**c**,**e**) before adsorption of Cd^2+^; (**b**,**d**,**f**) after adsorption of Cd^2+^ in the aqueous solution.

The test amendments were (1) a cement of 100 mass % ordinary Portland cement (OPC), (2) a cement of composition 80 mass % ordinary Portland cement + 20 mass % fly ash (FAC) and (3) mixture of 80 mass % ordinary Portland cement + 20 mass % zeolite (ZEC). Each of the OPC, FAC, and ZEC test components was taken in different polythene bags and mixed thoroughly with distilled water in order to have *w*/*s* = 0.5. The air was removed from plastic bags, and samples were hydrated at room temperature for two weeks. Then, the materials were manually crushed, grounded, and sieved at a size <0.075 mm. The crystalline structures of amendments were identified by X-ray diffraction (XRD, D8- Advance; Bruker, Germany) using Cu^2+^ Kα radiation at 2θ ranging from 5° to 80°. The morphology was characterized by scanning electron microscopy (SEM, S-3000N; Hitachi, Japan, operated at 30 kV). The structures and morphology of amendments examined by XRD and SEM are shown in Figure 1 and Figure 2.

### 2.2. Batch Adsorption Study in Solution

The batch method was performed to investigate the adsorption isotherms of Cd^2+^ on the three adsorbents. The different Cd^2+^ ion solutions used in the experiments were prepared by diluting a stock solution (1000 mg·g^−1^) with ultrapure water. During the experiment, the pH of the solution was adjusted by 1 M nitric acid or 1 M sodium hydroxide. To study the adsorption isotherm, 0.5 g adsorbents and 50 mL of Cd^2+^ solution (range of 40, 60, 80, 100, 120 mg·g^−1^) were mixed at the temperature of 25 °C for 24 h. Concentrations of Cd in the solutions were determined using a flame atomic absorption spectrometry (AAS; China, TAS-990 Super AFG, detection limit 0.03 μg·mL^−1^).

The removal efficiency (R, %) and adsorption capacity (*q_e_*, mg·g^−1^) were calculated from the following equations (Equations (1) and (2)), respectively:

(1) $$R=\left(C_{0}-C_{e}\right)/C_{0}\times 100$$

(2) $$q_{e}=V\times \frac{C_{0}-C_{e}}{m}$$

where *C*_0_ is the initial Cd^2+^ concentration (mg·L^−1^); *C_e_* is the concentration of heavy metal ions after adsorption (mg·L^−1^); V represents the volume (L) of Cd^2+^ solution; m is the dosage of the adsorbent (g).

In order to investigate the adsorption capacities of Cd^2+^, the most commonly used isotherm equations—namely, Langmuir (Equation (3)), Freundlich (Equation (4)), and Dubinin–Radushkevich (D–R) (Equation (5))—were applied to fit the experimental data. The expressions for these models, respectively are given as follows:

(3) $$\frac{C_{e}}{q_{e}}=\frac{1}{K_{L}q_{max}}+\frac{1}{q_{max}}C_{e}$$

(4) $$\mathrm{lg}q_{e}=\frac{1}{n}\mathrm{lg}C_{e}+\mathrm{lg}K_{F}$$

(5) $$\ln q_{e}=\ln q_{D}-\beta \times {\left[R\times T\times \ln\left(1+1/C_{e}\right)\right]}^{2}$$

where *q_max_* is the maximum adsorption capacity per unit weight of the adsorbent (mg·g^−1^), *K_L_* (L·mg^−1^) is the Langmuir constant, *K_F_* (L·g^−1^) is the Freundlich constant, n is the heterogeneity factor affected by temperature, β is the Dubinin–Radushkevich constant, q_D_ is the theoretical isotherm saturation capacity (mg·kg^−1^), R is gas constant (8.314 J·mol^−1^·K^−1^), and T is the absolute temperature (K). The mean free energy per molecule of adsorbate E (kJ·mol^−1^) was calculated by Equation (6).

(6) $$E=1/{\left(2\times \beta \right)}^{0.5}$$

The equilibrium factor (*R_L_*) of Langmuir isotherm can define the favorability and feasibility of the adsorption process, which is expressed in the following equation:

(7) $$R_{L}=\frac{1}{1+K_{L}C_{0}}$$

### 2.3. Preparation of Soil and the Incubation Experiment

Soil samples were collected from 0 to 20 cm depth of the heavy metal contaminated mining area, Dongfang city, Hainan Province, China. Dongfang has a history of mining activities, which led to Cd^2+^ contamination in the area. Mining activities have been completed. The surrounding environment is polluted by Cd^2+^ and needs restoration and remediation for agricultural practices and maximum crop production. The study area belongs to a tropical maritime monsoon climate, with an annual average temperature of 24 –25 °C, and an annual average rainfall of about 1100 mm. Stones, plant debris, and earthworms were removed from soil samples. The soil was air-dried for two weeks in the laboratory. Then, the soil samples were crushed and sieved to a particle size of <2 mm, and the basic physicochemical properties were analyzed [26]. Soil pH was measured in soil slurry with a soil–water ratio at 1:2.5 with an automated pH meter (SG2-ELK, Mettler Toledo). Soil cation exchange capacity (CEC) was determined by BaCl_2_-MgSO_4_ forced exchange method. The soil organic matter (SOM) concentration was determined by the potassium dichromate oxidation colorimetry method. The main characteristics of the soil are presented in Table 1. The concentrations of Cd^2+^ in soil ranged from 0.88 to 21.9 mg·kg^−1^ in China, according to the study on Cd^2+^ contaminated soil [27]. We prepared a simulated Cd^2+^ contaminated soil by using Cd^2+^ (NO_3_)_2_·4H_2_O to the air-dried soil samples (3.16 mg·kg^−1^) to achieve a Cd^2+^ concentration of 6 mg·kg^−1^. All soil samples were incubated in polyethylene plastic bags for 1 month maintaining 70% (*w*/*v*) moisture at 25 °C to reach equilibrium conditions between Cd^2+^ (NO_3_)_2_·4H_2_O and the soil.

Incubation experiments were carried out in 300 mL polythene plastic pots, with each pot containing 100 g of air-dried soil. The soil was then homogenously amended with OPC, FAC, and ZEC at a 1% (*w*/*w*) application rate, respectively. No amendment was used in the control soil (CK). Each treatment was repeated three times. All pots were covered with plastic lids that contained a hole to reduce water loss. During incubation equilibrium, pots were weighed weekly to maintain the moisture. All thermostatic chambers until equilibrium are reached (in the darkroom at room temperature for 1 month). Finally, the soil samples were air-dried and ground to pass through a <2 mm sieve for further analysis.

### 2.4. Leachability of Cd^2+^

To evaluate the effects on the availability, leachability, and speciation of Cd^2+^ in contaminated soil, the methods of DTPA extraction, toxicity characteristic leaching procedure (TCLP) extraction, sequential extraction procedure (BCR), and leaching column test were conducted.

#### 2.4.1. Sequential Extraction of Cd^2+^

BCR sequential extraction method was applied to study Cd fractionation [28]. Briefly, the acid-soluble/exchangeable fraction was extracted using 0.11 M acetic acid. The reducible fraction was extracted using 0.1 M hydroxylamine hydrochloride solution. The oxidizable proportion was extracted using 30% (mv^−1^) hydrogen peroxide and 1.0 M ammonium acetate. Then, the residual fraction was determined using three acid 3:1:1 mixture of (HCl-HNO_3_-HClO_4_). Finally, all the extracted samples were analyzed for Cd^2+^ using AAS.

#### 2.4.2. DTPA and TCLP Extraction

The bioavailability of Cd^2+^ in soil was detected by DTPA extraction method, following Lindsay and Norvell [29], as modified by ISO 14870 [30]. The extraction solution of DTPA was prepared by mixing 0.005 mol·L^−1^ DTPA, 0.01 mol·L^−1^ CaCl_2_, and 0.1 mol·L^−1^ triethanolamine (TEA), and pH was kept at 7.3 with 1 mol·L^−1^ HCl solution. Afterward, 10 g of samples (0.15 mm sieved) were added into a 20 mL volumetric flask with extraction solution and shaken for 2 h at room temperature. The suspensions were centrifuged at 4000 rpm for 10 min. The suspension was filtered by a 0.45 µm membrane. The Cd^2+^ in filtered supernatant was evaluated by flame atomic adsorption spectrometry (AAS, TAS-990 Super AFG, Chain). Cd^2+^ leaching toxicity was evaluated using the toxicity characteristic leaching procedure (TCLP), as described by Su et al. [31]. Then, 1.0 g of samples was mixed with 20 mL^−1^ of extraction solution (0.1 mol·L^−1^ CH_3_COOH, pH 2.88) in a 50 mL centrifuge tube, shaken for 18 h at 25 °C, and then centrifuged at 4000 rpm for 20 min. The resultant solution was filtered through a 0.45 µm filter membrane and analyzed for Cd^2+^ using AAS.

#### 2.4.3. Leaching Columns with SAR

To investigate the downward migration of Cd^2+^ from the treated and control soil under simulated acid rainfall, a leaching experiment was conducted. A PVC column with 3 cm in diameter and 15 cm in length, filled with (200.0 g) soil up to 10 cm length were used for each treatment and control (untreated soil). A layer of glass fiber and a two-centimeter height of quartz sand were placed on both the bottom and the top of all leaching columns to prevent soil particles to flow to the leachate. The leaching solution (simulated acid rain, SAR) was prepared by a diluted sulfuric (H_2_SO_4_)/nitric acid mixture (HNO_3_) (60/40 ww^−1^) with distilled deionized water until pH 4.0 ± 0.1. All columns were slowly saturated from the bottom with deionized water until they reached field capacity. The columns were stabilized for 48 h. Leaching experiments were conducted in triplicate for each treatment at room temperature (25 °C). The total amount of leaching was set at 6000 mL as standard. During the leaching process, 50 mL of leachate was collected consecutively as a test sample of the total amount of leached liquid at 120, 240, 500, 750, 1500, 2250, 3000, 3750, 4500, 5250, and 6000 mL. Each volume was allowed to fully drain from the soil column until the next volume was added. The leachates were filtered through a 0.45 µm membrane filter and analyzed for heavy metals concentration and pH values. Concentrations of Cd^2+^ in the leachate solutions were determined using AAS.

## 3. Results

### 3.1. Batch Adsorption Study

**Figure 3:**
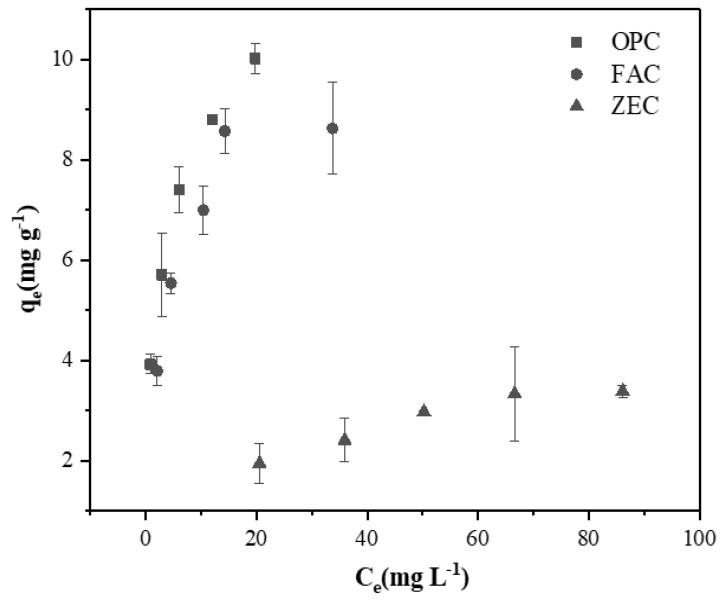
Adsorption isotherm of Cd by OPC, FAC, and ZEC pastes.

Significance differences were observed between the OPC, FAC, and ZEC before and after adsorption of Cd (Figure 1 and Figure 2). Figure 1a–c displays the XRD patterns of OPC, FAC, and ZEC before and after adsorption of Cd^2+^. Figure 2a–f shows the surface morphologies of OPC, FAC, and ZEC before and after adsorption of Cd^2+^. Figure 2a,c,e presents the images before adsorption of Cd^2+^, and Figure 2b,d,f presents the images after adsorption of Cd^2+^. The results of Cd^2+^ adsorption are shown in Figure 3, and their equation parameters are given in Table 2. Results revealed that the adsorption efficiency of OPC and FAC was 97.17% and 94.86% higher, respectively. Although the adsorption efficiency of ZEO was relatively low and reached 48.62%. According to the results, the Cd^2+^ adsorption capacity was prominently increased with the increasing rate of all adsorbents. However, the maximum adsorption capacity of Cd^2+^ in OPC, FAC, and ZEC were calculated as 10.97, 9.47, and 4.63 mg·g^−1^, respectively (Table 2).

### 3.2. Effects of Amendments on Soil pH and CEC

**Figure 4:**
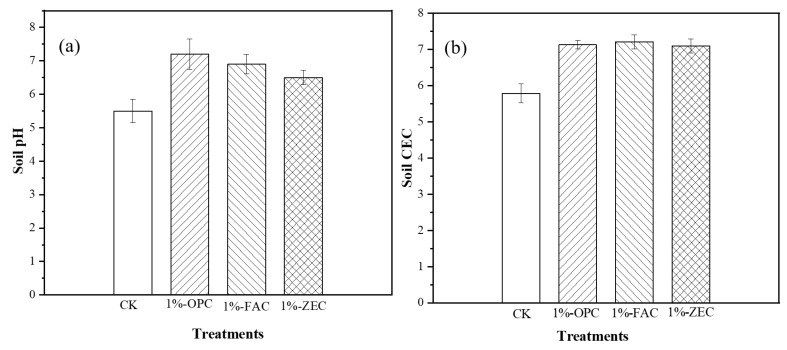
The changes of soil pH and CEC after treatment with amendments. Notes: (**a**) The changes of soil pH after treatment with 4 amendments; (**b**) The changes of soil CEC after treatment with 4 amendments. Treatments: control (CK), OPC paste, FAC paste, and ZEC paste.

The effects of the immobilizing agent on soil pH and CEC after 30 days incubation with OPC, FAC, and ZEC at three different amendments are presented in Figure 4a,b. The increase in soil pH was observed by 1.0 to 1.7 units with a 1% application rate, as compared to control soil (Figure 4a). Similarly, the addition of OPC, FAC, and ZEC prominently increased soil CEC, the increase observed at 1% OPC, FAC, and ZEC was 2.57–3.11 units higher, as compared to control (Figure 4b).

### 3.3. DTPA- and TCLP-Extractable Cd^2+^

**Figure 5:**
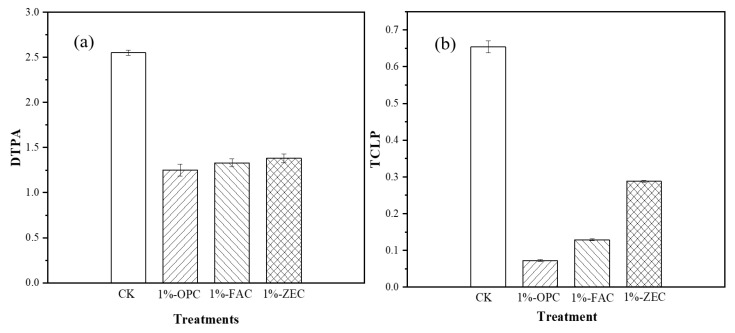
Amendments effect on Cd bioavailability and leachability. Notes: (**a**) Amendments effect on Cd extracted with DTPA; (**b**) Amendments effect on Cd TCLP. Treatments: control (CK), cement paste (OPC), fly ash cement (FAC) paste, and zeolite cement (ZEC) paste.

The availability of Cd^2+^ in soil affected by OPC, FAC, and ZEC was detected in DTPA extraction method. All added stabilizers showed prominent influence on DTPA-extractable Cd^2+^ in polluted soil, as shown in Figure 5a. The addition of OPC, FAC, and ZEC significantly reduced the DTPA-Cd^2+^ by 52, 48, and 46%, respectively, as compared to control. The effect of all amendments on solubility and leachability of Cd^2+^ after incubation is illustrated in Figure 5b. The concentration of Cd^2+^ in TCLP extract was significantly lower, following the addition of OPC, FAC, and ZEC. Specifically, the substantial decrease in TCLP Cd^2+^ was observed by 89.0% with OPC in soil, as compared to control. Addition of ZEC at 1% application was comparatively the least effective among all amendments, and decreased TCLP-Cd^2+^ by 56.0%, as compared to control. Application of FAC also performed better in reducing TCLP-Cd^2+^, and leaching was decreased by 80.3%, compared to control.

### 3.4. BCR Fraction of Soil Cd^2+^ after Incubation

**Figure 6:**
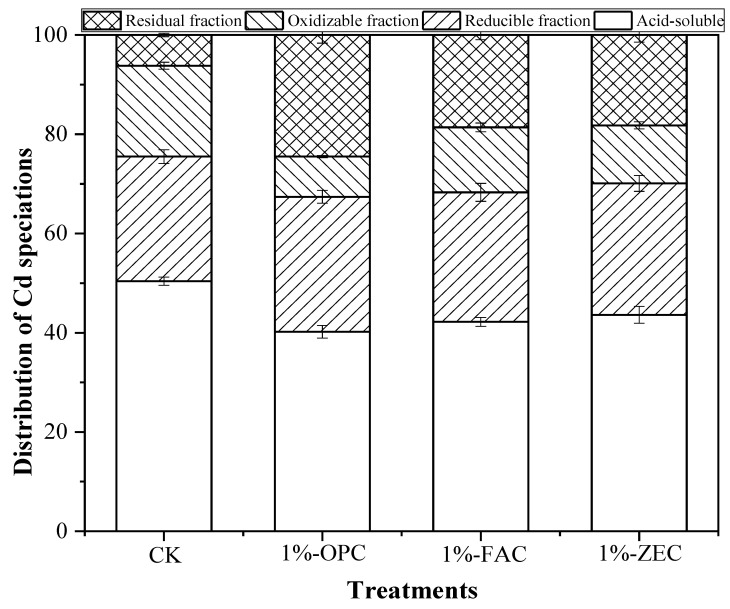
Amendments effect on Cd speciations. Treatments: control (CK), cement paste (OPC), fly ash cement (FAC) paste, and zeolite cement (ZEC) paste.

The portioning of Cd^2+^ was recorded among various geochemical fractionations in the contaminated soil after the addition of amendments. The exchangeable proportion of Cd^2+^ was reduced with the increasing addition of OPC, FAC, and ZEC (Figure 6). The reduction in the acid-soluble portion of soil Cd^2+^ occurred by 20.24, 16.27, and 13.49% for OPC, FAC, and ZEC, respectively, as compared to the control. Moreover, the percentage of reducible and oxidizable Cd^2+^ decreased with the increasing addition of OPC, FAC, and ZEC, relative to the control soil. The addition of all soil stabilizers also enhanced the Cd^2+^ residual proportion in soil. OPC, FAC, and ZEC enhanced the residual fraction of Cd^2+^ by 24.5, 18.6, and 18.2% with the addition at 1%, respectively, as compared to control.

### 3.5. Leaching Columns with SAR

#### 3.5.1. pH of the Leachate from the Columns Test

**Figure 7:**
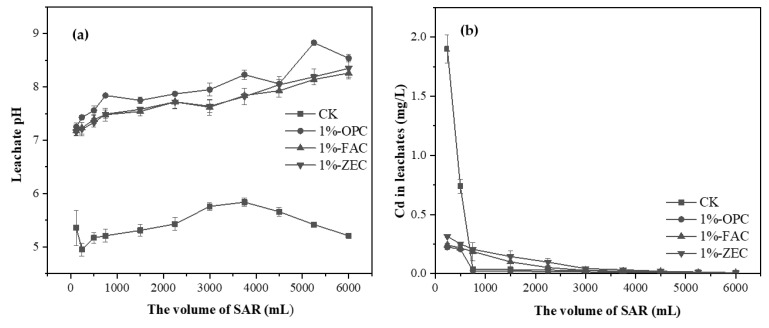
Characterization of leachate. Notes: (**a**) The leachate pH values leached with SAR (pH 4.0); (**b**) The leachate Cd concentrations leached with SAR (pH 4.0). Treatments: control (CK), cement paste (OPC), fly ash cement (FAC) paste, and zeolite cement (ZEC) paste.

Leachate pH values of untreated contaminated soil and treated soil mixture are presented in Figure 7. The initial pH for leached soil was 4.95. The pH value of the control leachate remained between 4.95 and 5.84 (Figure 7a). The application of OPC at 1% showed a significant increment in the soil pH, which increased by 3.59 units, compared with the initial soil pH (Figure 7a). Similarly, in the case of FAC at 1%, the pH of the leachate columns increased by 3.31 units (Figure 7a). A similar pattern (Figure 7a) was observed with respect to the leachate soil pH values in ZEC treatments. The addition of ZEC offered a significant increase in soil pH by 3.40 units. The leachate soil pH values were positively correlated with the addition of the three materials. Thus, with the continuous hydrates of mortar waste, the pH of leachate controlled by OPC, FAC, and ZEC were generally found at a high level and remained always higher than the control soil.

#### 3.5.2. Cd^2+^ Concentration in the Leachate

Figure 7b shows the change in Cd^2+^ concentration in the leachate as a function of leaching solution volume. From the leaching experiments under SAR, the Cd^2+^ leaching showed a linearly decreasing trend. Effluent Cd^2+^ from control columns initially decreased from 1.9 to 0.04 mg·g^−1^ during the collection of the first 1 L and then stabilized at 0.01 mg g^−1^ during the collection of the final 5 L (Figure 7b). OPC addition decreased the Cd^2+^ concentration notably from 0.22 to 0.03 mg·g^−1^ during the collection of the first 1 L and then reached the levels of 0.01 mg·g^−1^ after the collection of the last 5 L (Figure 7b). The Cd^2+^ concentration in the FAC-treated columns decreased from 0.24 to 0.19 mg·g^−1^ during the collection of the first 1 L. Afterward, Cd^2+^ concentrations were sustained at 0.09 mg·g^−1^ during the collection of the last 5 L (Figure 7b). Similar to OPC and FAC, the concentrations of Cd^2+^ in ZEC decreased significantly from 0.32 to 0.21 mg·g^−1^ during the collection of the first 1 L. Furthermore, ZEC addition kept Cd within a concentration of 0.15 mg·g^−1^ as the remaining 5 L was collected. Compared to the CK-soil, there was a significant reduction in Cd^2+^ leachability in soil treated with cement-based demolition waste. As shown in Figure 7b, the total amount of Cd^2+^ in the CK–soil in the column was 2.82 mg·g^−1^, and that of the waste-cement-treated soil column was significantly decreased to 0.55, 0.89, and 1.14 mg·g^−1^, respectively, during the SAR leaching test.

## 4. Discussion

### 4.1. Cd^2+^ Adsorption Mechanism

The Cd^2+^ adsorption isotherm was observed at different initial heavy metals concentrations from 40 to 120 mg g^−1^. The coefficients of Langmuir and Freundlich equations along with R^2^ (Table 2), obviously indicated that both equations were appropriately fitted (R^2^ greater than 0.9) for our data. A value of *n* (the heterogeneity factor) greater than 1 indicated the heterogeneous adsorption and favorable conditions for adsorption [32]. The R_L_ value greater than 1 represents unfavorable adsorption, and R_L_ equal to 1 indicates linear adsorption isotherm, while R_L_ value lower than 1 represents favorable adsorption [33]. When R_L_ = 0, it is irreversible adsorption. The value of R_L_ was calculated between 0.056 and 0.423, implying that the adsorption of Cd^2+^ on the OPC, FAC and ZEC was favorable (R_L_ < 1). Under these experimental parameters, it can be concluded that favorable adsorption conditions were provided, which led to the heterogeneity of the adsorption surface.

There are two possible mechanisms that may be accountable for the Cd^2+^ adsorption by waste cement: (a) specific adsorption or chemical adsorption, such as complexation reaction, which is selective, irreversible, and monolayer; (b) non-specific adsorption, mostly non-selective, reversible and monolayer or multi-molecular layer [23]. The mean free energies of adsorption E can be used to determine the adsorption type. If this value is less than 8 kJ·mol^−1^, the adsorption indicates a physical process, while at 8–16 kJ·mol^−1^, the adsorption indicates a chemical process. In this work, the E values for sorption of Cd^2+^ ions on the waste cement adsorbents were about 0.02–3.5 kJ·mol^−1^, which indicated that adsorbents implicate the physisorption mechanism [34]. According to Balaz et al. [35], the average free energy was lower than 8 kJ·mol^−1^, which was dominated by physical adsorption, resulting in low average free energy, but chemical adsorption with new compounds may exist in the adsorption process and does not play a leading role. However, based on our XRD results, the three materials have complex surfaces containing several functional groups, and the adsorption on the complex surface can occur through a variety of adsorption mechanisms.

Slight differences in mineral composition were observed for all tested materials, as shown by XRD analysis. The main mineral phases of each material were similar. Figure 1 revealed that before adsorption of Cd, the main mineral phases of the three materials were CaCO_3_ and SiO_2_ or silicate compounds. However, after adsorption of Cd^2+^, the diffraction peak of silica or silicic acid compounds disappeared or weakened, while the diffraction peak of carbonate was weakened. Additionally, high CO_3_^2−^ permitted precipitation of CdCO_3_ in the Cd^2+^ adsorbed on the waste cement. In addition, the three materials with high SiO_2_ content would certainly cause higher available Si content in the solution. Higher Si concentration in the solution could alleviate heavy metal toxicity by co-deposition of metals with silicates [36]. Therefore, adsorption of Cd^2+^ on the waste cement was ascribed not merely to physical adsorption; chemical adsorption of precipitation of Cd^2+^, particularly Cd^2+^ with CO_3_^2−^ and Si on the waste cement, also played a role.

The scanning electron microscope (SEM) micrographs before and after adsorption of Cd^2+^ revealed that before adsorption of Cd^2+^, the surface morphology and structure of the three materials were homogeneous and smoother and had more pores. There was a prominent change in the surface morphology of the three materials after adsorption of Cd^2+^, and surfaces became more irregular and coarser. From our SEM results, we can suggest that the addition of the three studied materials could increase Cd^2+^ immobilization through adsorption and precipitation.

### 4.2. How Different Soil Amendments Change Soil pH and CEC

In the present study, among all the amendments, OPC, FAC and ZEC have a significant effect on soil pH owing to their liming effects and high alkalinity [37]. The high pH values of OPC, FAC, and ZEC were derived from the dissolution of cement, which is the major component of cement-based demolition waste [38]. Generally, OH^−1^ and a plant of Ca^2+^ and Si^2+^ contained in materials can neutralize and replace H^+^ in soil solution and increase soil pH [39]. During continuous hydrolysis of cement, cement generated alkaline substances (Ca(OH)_2_, CaCO_3_, C-S-H, and SiO_2_ gel). According to the results of this study, OPC, FAC, and ZEC have high pH, ash contents, and plenty of various surface functional groups (Figure 3) on the surface could cause an increment in soil pH. These results were consistent with Yuan et al. [40], who argued that an increase in pH can be explained by increases in Ca^2+^ contents ash content, carbonate, and several additional surface functional groups on materials. Meanwhile, waste cement has high pH and Ca^2+^ contents due to the presence of greater concentrations of calcium carbonate and silicate ions in the materials, which could increase soil pH and promote metal carbonate through precipitation [41].

CEC is a dominant factor for metal immobilization. Soil CEC reflects the number of negative charges on the material surface, which can be balanced by exchangeable cations [42]. Enhanced values of soil CEC exhibited the development of more negative charge in the treated soils. This increment could be attributed to the presence of oxygen-containing functional groups such as silicates and carbonates in the OPC, FAC, and ZEC, which have a high affinity for divalent Cd ions. In addition, the addition of OPC, FAC, and ZEC increased soil CEC, which could lead to an increase in ion exchange and adsorption of Cd^2+^ on CaCO_3_. Furthermore, OPC, FAC, and ZEC contain appreciable amounts of inorganic constituents (Ca^2+^, Mg^2+^, etc.), which are released into the soil, thereby promoting soil CEC and providing available nutrients for plant growth [43].

### 4.3. Impact of Amendments on Cd^2+^ Fractions

Application of OPC, FAC, and ZEC modified the concentration of Cd^2+^ in various fractions. Particularly, values varied with the nature of amendments. In general, high CEC and pH value and surface functional groups of the materials may have the ability to reduce Cd toxicity in contaminated soil and increase its stabilization. The influence of OPC, FAC, and ZEC on Cd^2+^ immobilization was observed, and in particular, the three materials could decrease the acid-soluble/exchangeable proportion of Cd^2+^ and increase the residual fraction in polluted soil. These results are in accordance with the observation of Zhang et al. [44], who suggested that heavy metal soluble form reduced with the increase in soil pH. The hydrolysis and dissolution of alkaline substances on OPC, FAC, and ZEC surface could increase soil pH and induce Cd^2+^ precipitation as Cd^2+^ oxy(hydroxides) and CdCO_3_, which is one of the mechanisms of metals stabilization, as stated by Park et al., Jiang et al. and Ahmad et al. [42,45,46]. Furthermore, the activity of free Cd^2+^ ions decreases at high pH; therefore, the increased values of pH might have led to the decreased activity of free metals, thereby reducing the available fraction of the metals.

In the present study, the three materials led to an increase in residual Cd^2+^ fraction, which might have occurred due to adsorption and precipitation. Moreover, McLean and Bledsoe [47] observed alkaline conditions were necessary for the formation of trace metal hydroxides, oxides, carbonate, and phosphate precipitates, which limit their dissolution, and many negative sites adsorption cations are also generated on soil materials. Waste cement has great potential to stabilize heavy metal contamination due to high pH and very high ANC values resulting from the hydration products of the cement paste and perform better for heavy metal immobilization in acidic polluted soil [23].

### 4.4. Impact of Amendments on Cd^2+^ Solubility and Bioavailability

Waste cement readily transforms heavy metals from the bioavailable portion to a more stable residual form; therefore, heavy metals mobility is significantly reduced [28]. The DTPA- and TCLP-extractable concentrations of Cd in the soil were greatly immobilized by all amendments over the one-month incubation period. The reduction efficiency of the solubility and bioavailability of Cd^2+^ might be due to higher alkalinity, which is induced by the continuation of cement hydration, Ca^2+^ ions, total CaCO_3_, silicate [48], and various functional groups on the surface. The significant decline (*p* < 0.05) in the DTPA- and TCLP-extractable Cd^2+^ was observed with increasing OPC, FAC, and ZEC rates. Moreover, high alkalinity, Si contents [48], and total CaCO_3_ on the waste cement might be a significant underlying reason for Cd^2+^ immobilization in highly polluted soil. Specifically, cement potentially reduced heavy metal leachability in TCLP extraction. Our findings are in line with Xi et al. [49], who suggested that the addition of cement substantially reduced TCLP-extractable heavy metal because of high adsorption ability on the cement surface with an increase in soil pH.

### 4.5. Changes in the Leachate pH and Cd^2+^ Concentration

Generally, the pH of leachate in the control soil decreased at the initial stage (0–1 L), then increased slightly afterward (2–4 L), and finally gradually reduced to 4–6 L. Tabatabai and Olsontsw [50] pointed out that the buffering mechanism of soil against acid rain is mainly the exchange of base ions (K^+^, Na^+^, Ca^2+^, and Mg^2+^) with H^+^ and the weathering of soil minerals (Fe^3+^, Al^3+^, and Si^4+^). In the initial stage of leaching (0–1 L), a significant number of base ions were leached, and the value of the leached pH decreased rapidly with the leaching of high-intensity acid rain. When the value of leachate pH reached the lowest, the weathering mechanism of soil minerals predominated, that is, the acid hydrolysis of primary and secondary aluminosilicate in soil, accompanied by the release of a small number of base ions, which compensated for the leaching loss of soluble and exchangeable base ions. This mechanism has a strong buffering ability but a slow kinetic response, leading to the rise of leachate pH. However, with continuous leaching, the mineral hydrolysis weakened, results showed a slight decrease in the leachate pH after 4 L.

Compared with the control, the addition of the waste cement significantly increased the buffering capacity of soil against acid rain, and the leachate pH increased by 3.31–3.59 units in 1–6 L leaching, respectively. There were also significant differences in leachate pH in all amendment–soil systems. Longer leaching time tends to increase the leachate pH; in addition, the values were maintained at a high level over time, because waste cement products are alkaline substances, and SAR promoted the dissolution of waste cement. The hydrolyzed OH^−^ from cement neutralizes the H^+^ in SAR and consumes the acid in the soil, thus increasing the leachate pH. At the same time, alkaline substrates (CaCO_3_, C-S-H, SiO_2_ gel) were produced due to the dissolution of waste cement, which can also neutralize H^+^ in acid rain.

The content of Cd^2+^ in the control soil was the highest, indicating that acid rain promoted the release of Cd^2+^ in the soil, improved the activity of Cd^2+^ in the soil, and was easy to be leached [51]. The leached concentrations of Cd^2+^ were significantly lower in cement-applied than in the control. The addition of waste cement reduced the content of Cd^2+^ in the leachate, and there was an obvious trend of reduction in the whole process of leaching (1–6 L) (Figure 7b). The content of Cd^2+^ in leached solution was at a low level and gradually decreased due to the following reasons. Firstly, the increase in soil pH, caused by the addition of the alkaline waste cement, may benefit the residue of Cd^2+^ in soil. As Todaro et al. [52] and Sauvé et al. [53] indicated, the pH value is one of the main factors affecting the mobility of metals from contaminated heavy metals. The higher soil pH is essential to form perceptible metal such as metal hydroxide, oxide, carbonate, and phosphate precipitates, as well as increased the negative sites adsorbing cations on the soil surface, leading to heavy metal adsorption onto the soil surface [54]. Previous studies [55,56] reported that soil alkalinity controls the release and migration of Cd^2+^ in soil, which is the main condition for Cd^2+^ immobilization. Secondly, waste cement contained a large number of mineral compositions such as CaCO_3_, C-S-H, and SiO_2_ gel, etc., which promoted the direct formation of cadmium carbonate precipitation, cadmium phosphate precipitates, etc. The treatment of waste cement converted partial cadmium ions into insoluble states, which enhance cadmium immobilization in soil. Finally, the high SiO_2_ content of the waste cement amendment would inevitably lead to the high content of available Si in soil. Application of exogenous silicon could alleviate heavy metal toxicity through co-deposition of Cd^2+^ with silicate [36,57]. Therefore, the high concentration of Si in the waste cement could reduce the mobility of Cd^2+^ in the soil. However, the addition of waste cement significantly reduced the content of Cd^2+^ in the leachate, compared to the control, and the accumulated Cd^2+^ in the treated columns was much higher than the level V (0.01 mg·g^−1^) of the Chinese National Quality Standard for Surface Water (GB 3838-2002). This indicated that the application of waste cement with a mass ratio of 1% to moderate and severe Cd^2+^ pollution in mining soil may continue to pose a risk of Cd^2+^ pollution to the surrounding water. Therefore, supplementary trials on waste cement should be carried out in longer terms, under field conditions, and using different levels of application to remediate multi-metals in polluted soil.

### 4.6. Mechanism of Waste Cement on Cd^2+^ Immobilization

The purpose of this study was to assess the efficacy of different waste cement commonly used in construction materials including cement, fly ash cement, and zeolite cement, in order to quantify their effectiveness for the adsorption of Cd^2+^ in solution and also bioavailability, mobility, and leaching of Cd in soil. All amendments showed significant increment in soil pH due to their higher alkalinity and high ANC [23]. Waste cement has high pH, Ca^2+^, and Si contents due to the continuous hydrolysis of cement and the presence of a greater amount of calcium carbonates and silicate in the waste cement, which could increase soil pH and promote metal hydroxide, oxide, carbonate, and phosphate through precipitation [23]. The hydrolysis and dissolution of these alkaline substances could increase soil pH and induce Cd^2+^ precipitation [58]. The DTPA-extractable and TCLP-extractable concentration of Cd^2+^ in the soil was greatly immobilized by all amendments over one month incubation period. The great efficiency of waste cement in reducing the solubility and leachability of Cd^2+^ might be possible due to the higher alkalinity induced by hydrolysis of cement, Ca^2+^ ions, and CaCO_3_. Therefore, waste cement increased soil pH by about 1.0–1.7 units, which might reduce Cd^2+^ solubility and leachability through precipitation and adsorption [12]. Moreover, the addition of waste cement in acidic soil could increase soil pH, which might cause heavy metal immobilization through precipitation and adsorption. The reduction in Cd solubility might be due to high pH and the presence of a wide range of carbonate and silicon content of the material, which could cause the greatest decline in the availability of heavy metals through precipitation and co-deposition on soil [48,59]. The impact of waste cement on Cd^2+^ transformation from its bioavailable fraction to residual form was observed through BCR analysis.

## 5. Conclusions

This study investigated the efficiency of waste cement for adsorption of Cd^2+^ from aqueous solution and immobilization of Cd^2+^ from contaminated soil. The Langmuir adsorption isotherm showed adsorption capacity of 10.97, 9.47, and 4.63 mg·g^−1^. XRD results exhibited the mineral formation of Cd^2+^ after precipitation, and SEM results also revealed clear differences between pre- and post- Cd^2+^ adsorption. It is suggested that waste cement could be considered as an alternative for Cd^2+^ removal from polluted water. At the same time, this study also revealed that unusable waste cement had a great effect on in situ Cd^2+^ immobilization by using DTPA and TCLP extraction techniques. This could have a major contribution to reducing Cd^2+^ concentration in the acid-soluble proportion and increasing the percentage of Cd^2+^ in the residual fraction. The addition of waste cement increase soil pH, which might contribute to enhanced Cd^2+^ adsorption and precipitation. In addition, the column experiments confirmed that the Cd^2+^ in the leachate was inhibited by waste cement treatments. Under simulated acid rain conditions, the amendments had positive effects on Cd^2+^ immobilization, thus reducing the leaching risk and mobility of Cd^2+^ to the environment. Furthermore, our results might contribute to the scientific solutions to immobilizing Cd^2+^ by emerging low-cost alkaline amendments. In conclusion, further study is needed to evaluate the immobilization effect of these amendments on heavy metals in multi-polluted soil.

## Figures and Tables

**Table 1:** Selected physicochemical properties of the contaminated soil.

Physicochemical Properties	Contaminated Soil
Water content (%)	23.37
pH	5.5
Organic matter (g·kg^−1^)	9.965
Cation exchange capacity (CEC) (cmol·kg^−1^)	5.79
Cd (mg·kg^−1^)	3.16

**Table 2:** The parameters for adsorption of Cd in OPC, FAC, and ZEC obtained from various adsorption models.

Material	OPC	FAC	ZEC
Langmuir			
K_L_ (L·mg^−1^)	0.341	0.421	0.034
q_max_ (mg·g^−1^)	10.97	9.47	4.63
R^2^	0.994	0.99	0.983
Freundlich			
K_F_	4.177	3.325	0.379
*n*	3.340	3.262	1.968
R^2^	0.996	0.876	0.897
Dubinin–Radushkevich model			
q_D_ (mg·g^−1^)	8.182	7.897	3.321
E (kJ·mol^−1^)	3.5	0.56	0.02
R^2^	0.788	0.889	0.908

Note: OPC: ordinary Portland cement, FAC: fly ash cement, ZEC: zeolite cement.

## Data Availability

Not applicable.
